# Cotrimoxazole Prophylaxis and Tuberculosis Risk among People Living with HIV

**DOI:** 10.1371/journal.pone.0083750

**Published:** 2014-01-08

**Authors:** Christopher J. Hoffmann, Richard E. Chaisson, Neil A. Martinson

**Affiliations:** 1 Johns Hopkins University School of Medicine and Center for TB Research, Baltimore, Maryland, United States of America; 2 Perinatal HIV Research Unit, Johannesburg, South Africa; University of Cape Town, South Africa

## Abstract

**Objectives:**

Many randomized and cohort studies have reported a survival benefit with cotrimoxazole prophylaxis without detecting a difference in tuberculosis (TB) incidence by cotrimoxazole status. However, several *in vitro* studies have reported that cotrimoxazole possesses anti-TB activity. We sought to compare TB incidence and TB diagnostic yield by cotrimoxazole use among participants in a well characterized cohort of HIV-infected adults living in a high TB prevalence region.

**Methods:**

We analyzed prospective data from a long-term longitudinal cohort of adults receiving HIV care and TB investigations in Soweto, South Africa. Using longitudinal analysis, we compared total and laboratory confirmed TB incidence by cotrimoxazole status as well as all-cause mortality. In addition, we compared TB culture results by cotrimoxazole status.

**Results:**

In a multivariable analysis, adjusted for sex, body mass index, WHO clinical stage, time-updated CD4 count, and antiretroviral therapy status, we observed an association between cotrimoxazole and an increase in TB incidence (hazard ratio 1.7, 95% CI: 1.2, 2.2). However, when restricted to laboratory-confirmed TB, there was no association between cotrimoxazole and TB incidence (hazard ratio: 0.97, 95% CI: 0.39, 2.4). In TB cases, we found no difference in the proportion of positive sputum cultures or days to culture positivity by cotrimoxazole status. Cotrimoxazole was associated with a reduction in mortality.

**Conclusions:**

In this cohort with a mortality benefit from cotrimoxazole, we found an increased risk of all TB among individuals using cotrimoxazole, likely a result of residual confounding, but no association between use of cotrimoxazole and laboratory-confirmed TB. Cotrimoxazole did not compromise TB diagnosis.

## Background

Tuberculosis (TB) is the leading cause of death among people with HIV in Africa [1], [2]. Owing to the immense number of patients receiving TB treatment, even a small reduction in risk of TB disease among HIV-infected individuals could have important effects on the TB epidemic [3]. Cotrimoxazole prophylaxis is recommended for use by the World Health Organization (WHO) to reduce morbidity and mortality among individuals with TB or advanced HIV disease [4]. *In vitro* data have suggested that cotrimoxazole has activity against *Mycobacterium tuberculosis* [5]–[7]; yet studies of cotrimoxazole prophylaxis in Africa have not demonstrated an effect on TB incidence [8], [9]. In addition to the potential of reducing TB incidence or mortality, of theoretical concern is that anti-mycobacterial activity of cotrimoxazole could make detection of TB more difficult by reducing the proportion of TB patients with smear positive disease, or prolonging time to mycobacterial culture positivity. To test the hypotheses that cotrimoxazole either reduces TB disease incidence or confounds TB diagnosis, we assessed TB incidence and culture characteristics in an HIV cohort in a high TB prevalence setting in South Africa.

## Methods

This research was conducted according to the principles expressed in the Declaration of Helsinki; written informed consent was obtained from all participants prior to study procedures and the study was approved by institutional review boards of the University of the Witwatersrand and Johns Hopkins University School of Medicine. Participants were aged ≥18 years with CD4 counts <350 cells/mm^3^ and enrolled in a prospective cohort of HIV-infected individuals in Soweto, South Africa [10], [11]. Participants had scheduled clinic visits every 6 months but were also assessed at unscheduled visits for acute illnesses. Assessment at enrollment and all subsequent visits included a routine symptom-based assessment for TB with laboratory investigations as indicated. Cotrimoxazole was prescribed according to modified WHO guidelines and isoniazid preventive therapy was offered to participants with positive tuberculin skin tests and, for a short period, to all participants irrespective of their tuberculin skin test results. Participants meeting South African national antiretroviral therapy (ART) initiation criteria were referred for treatment. Sputum was obtained for smear microscopy and mycobacterial culture using the MGIT system (Becton, Dickinson, and Company, Franklin Lakes, New Jersey, USA). Laboratory and clinical records were sought for all participants initiated on TB therapy at other clinics. Patients were excluded if they were diagnosed with TB within 60 days of study entry as TB diagnosed during this period was assumed to be prevalent disease.

TB disease was defined as *clinically diagnosed* if TB treatment was started in the absence of laboratory confirmation, and *laboratory confirmed* if there was either detection of acid-fast bacilli on sputum smear or fine needle aspirate, or a biopsy suggestive of tuberculosis, or detection of *M. tuberculosis* in culture media from any site. If cotrimoxazole was initiated in the 60 days prior to a diagnosis of TB, we categorized the TB episode as not exposed to cotrimoxazole because cotrimoxazole may have been started as a result of TB symptoms but prior to the TB diagnosis. Body mass index (BMI) was categorized according to the WHO International Classification of Adult BMI into underweight and normal weight (BMI <25 kg/m^2^) or overweight and obese (BMI ≥25 kg/m^2^). This dichotomous classification of BMI was selected after preliminary analysis identified similar risk in TB diagnosis between underweight and normal weight patients.

Multiple imputations, using a repeated-imputation inference, were used to assign values for the 16% of participants with missing values for BMI and the 8% of participants with missing values for WHO stage. We used logistic regression models for imputation with BMI and WHO stage both classified as dichotomous variables and assumed that data were missing completely at random. Eight separate imputed datasets were created and combined using Rubin’s combination rules to form one set of results [12].

We used Cox proportional hazards modeling to assess for potential associations with incident TB; we repeated the analysis restricting it to laboratory confirmed TB. We repeated the analysis using the outcome of mortality as a method to verify cotrimoxazole use in this population by comparing our results with the known reduction in mortality from the published literature. CD4 count was transformed by taking the square root, to approximate a normal distribution, and used as a time-updated variable. ART and cotrimoxazole use were similarly used as a time-updated variables. We did not use time-updated WHO clinical stage and BMI due to limitations in follow-up data for these two variables. Factors with a p<0.1 or with a clinically plausible association were included in a multivariable model. Time to culture positivity was compared using the Wilcoxon rank sum test. Proportions were compared using the chi-square test. Stata/MP 13.0 was used for all analyses (StataCorp LP, College Station, Texas, USA).

## Results

Of 2590 participants followed between April 2003 and December 2009, 198 were excluded due to prevalent TB at the time of enrollment. Among the remaining 2393 participants, the median age was 33 years (interquartile range [IQR]: 29, 39), 686 (29%) had WHO clinical stage 3 or 4 disease, and the median CD4 count at entry was 209 cells/mm^3^ (IQR: 115, 292); 1,846 (77%) were women (Table 1). A total of 4,875 person-years of follow-up time were included in these analyses. Cotrimoxazole was prescribed to 1,294 cohort participants (54%) for a total of 688 person years, with a median CD4 count at cotrimoxazole initiation of 162 cells/mm^3^ (IQR: 97, 257).

**Table 1:** Participant characteristics (2,393).

	N (%) or median (IQR)
Sex, Female	1,846 (77)
Age, median years	33 (29, 39)
BMI, kg/m^2^	
<25	1,170 (49)
≥25	834 (35)
Missing	389 (16)
WHO clinical stage	
1 or 2	1,513 (63)
3 or 4	686 (29)
Missing	194 (8)
CD4 at cohort entry, cells/mm^3^	
Median	209 (115, 292)
ART at any time during the study	
No	1,635 (68)
Yes	758 (32)

During follow-up, 179 participants (7.5%) were diagnosed with incident TB (either based on laboratory findings or clinical suspicion). The overall rate was 3.7 cases per 100 person-years (95% confidence interval [CI]: 3.2, 4.2) with rates of 7.6 (95% CI: 5.8, 9.9) and 3.0 (95% CI: 2.5, 3.6) per 100 person-years among patients receiving and not receiving cotrimoxazole, respectively (p <0.01). In multivariable analysis, factors associated with incident TB were male sex, lower BMI, more advanced WHO clinical stage at cohort entry, lower CD4 count, not receiving ART, and receipt of cotrimoxazole (hazard ratio [HR]: 1.7, 95% CI: 1.2, 2.2; Table 2).

**Table 2:** Characteristics associated with all TB, laboratory confirmed TB, and mortality.

	Tuberculosis				Mortality
	All TB		Laboratory confirmed TB		
	Univariable hazard ratio	Multivariable hazard ratio	Univariable hazard ratio	Multivariable hazard ratio	Multivariable hazard ratio
	(95% confidence interval)	(95% confidence interval)	(95% confidence interval)	(95% confidence interval)	(95% confidence interval)
Cotrimoxazole					
No	Referent, p<0.01	Referent, p<0.01	Referent, p<0.01	Referent, p = 0.9	Referent, p = 0.09
Yes	2.8 (2.2, 3.5)	1.7 (1.2, 2.2)	2.9 (1.9, 4.4)	0.97 (0.39, 2.4)	0.48 (0.21, 1.1)
Sex					
Male	Referent<0.4	Referent, p = 0.04	Referent, p = 0.3	Referent, p = 0.1	Referent, p = 0.9
Female	0.90 (0.570, 1.1)	0.73 (0.54, 0.98)	0.80 (0.53, 1.2)	0.58 (0.28, 1.2)	0.98 (0.59, 1.6)
BMI, kg/m^2^					
<25	p<0.01	Referent, p<0.01	Referent, p<0.01	Referent, p = 0.8	Referent, p = 0.01
≥25	0.52 (0.36, 0.74)	0.54 (0.36, 0.80)	0.51 (0.32, 0.80)	0.93 (0.44, 2.0)	0.34 (0.16, 0.70)
WHO clinical stage					
1 or 2	Referent p <0.01	Referent p<0.01	Referent, p = 0.09	Referent, p = 0.1	Referent, p = 0.4
3 or 4	1.8 (1.4, 2.2)	1.6 (1.3, 2.1)	1.4 (0.94, 2.0)	1.8 (0.89, 3.5)	1.2 (0.79, 1.9)
Square root CD4, per 1 unit increase	0.94 (0.91, 0.96), p<0.01	0.93 (0.90, 0.95), p<0.01	0.93 (0.88, 0.97), p<0.01	0.88 (0.82, 0.96), p<0.01	0.87 (0.81, 0.94), p<0.01
Antiretroviral therapy					
No	Referent, p = 0.05	Referent, p = 0.01	Referent, p<0.01	-	Referent, p<0.01
Yes	0.73 (0.54, 0.99)	0.64 (0.45, 0.90)	0.04 (0.005, 0.27)	-	0.30 (0.17, 0.53)

TB laboratory investigations were conducted for 665 participants, including 52 diagnosed with culture confirmed incident TB. In an adjusted analysis, when restricting to culture confirmed TB and including potential confounders of sex, BMI, WHO clinical stage, CD4 count, and ART receipt, we found no association between cotrimoxazole use and incident TB (HR: 0.97; 95% CI 0.39, 2.4; Table 2).

In assessing TB diagnostic yield by cotrimoxazole status, a similar proportion of TB cultures were positive among patients receiving and not receiving cotrimoxazole (14/170 [8.2%] and 40/397 [10%], respectively, p = 0.5); a similar proportion of sputum smears were also positive by fluorescence microscopy 8/206 (3.9%) and 21/525 (4.0%), respectively (p  =  0.9). Time to mycobacterial culture positivity in MGIT was also similar with a median time to positive culture of 20 days (IQR: 16, 23) and 18 days (IQR: 14, 24), respectively, for patients receiving and not receiving cotrimoxazole (Wilcoxon rank sum p value for difference  =  0.2).

During follow-up 125 participants died. In univariable Cox proportional hazards modeling, death was associated with cotrimoxazole use, lower CD4 count, male sex, higher WHO clinical stage, lower BMI, and not receiving ART (results not shown). In multivariable analysis, including cotrimoxazole, and factors significant in univariable analyses, the effect size for cotrimoxazole shifted to reduced mortality (HR 0.48, 95% CI: 0.21, 1.1; Table 2).

## Discussion

We identified neither a protective effect on TB incidence nor an apparent effect on the diagnosis of TB among HIV-infected patients receiving cotrimoxazole. Unexpectedly, we found that the risk of TB disease appeared to be increased among individuals who were receiving cotrimoxazole. Although this effect persisted after adjusting for CD4 count and WHO clinical stage, we believe that this was a result of residual confounding, a hypothesis supported by the loss of association when we restricted our analysis to culture confirmed TB. A clinical finding of no reduction in TB incidence by cotrimoxazole status is consistent with results of prior studies of cotrimoxazole. One of the early randomized trials of cotrimoxazole among HIV-infected individuals reported 22 cases of TB among 271 participants in the placebo group and 17 cases among 270 participants in the cotrimoxazole group (p = 0.6) [9]. Another large trial with over 200 cases of incident TB, also found no difference in TB incidence among participants who did and did not receive cotrimoxazole [8]. Importantly, our effect size related to cotrimoxazole and mortality was consistent with other studies [13]–[18]. However, we acknowledge a lack of statistical significance with this result, likely due to the limited power of this analysis due to relatively few deaths.

While these results support prior findings of no reduction in TB incidence with once-daily cotrimoxazole prophylaxis and add new information that cotrimoxazole prophylaxis does not confound laboratory diagnosis of TB, it is unclear what effect higher doses of cotrimoxazole would have on TB incidence or diagnosis [5]–[7], [19]. However, it would appear that pulmonary cotrimoxazole concentrations with prophylaxis dosing are higher than the concentrations used for *in vitro* testing, thus it is unlikely that a higher dose would change the effect on TB incidence [19].

This study has the strengths of a well characterized and prospectively followed cohort. However there are several important limitations, which include lack of TB laboratory data on half of TB cases (because patients received empiric therapy). Another limitation is that additional considerations beyond CD4 count may have influenced cotrimoxazole provision; these factors may have been associated with TB risk and chance of TB evaluation or treatment, leading to residual confounding and causing a spurious association between cotrimoxazole and an increased risk of TB. We believe the hypothesis of residual confounding is supported by the attenuation in effect size when we limited the model to laboratory confirmed cases.

Cotrimoxazole is a vital part of the HIV care package with well documented improvements in survival. However, cotrimoxazole prophylaxis does not appear to affect either TB disease incidence or detection.
